# Severe Macrocytic Anemia in a Young Alcoholic Male: A Case Report

**DOI:** 10.7759/cureus.91350

**Published:** 2025-08-31

**Authors:** Inês Ferreira, Inês Fiúza M. Rua, Diogo Ramos, Sérgio Cabaço, André Valente

**Affiliations:** 1 Internal Medicine, Unidade Local de Saúde São José, Lisbon, PRT

**Keywords:** alcohol use disorder, folate, macrocytic anaemia, megaloblastic anaemia, pancytopenia, vitamin b12

## Abstract

Vitamin B12 and folate deficiency can lead to severe macrocytic anemia, apart from other consequences such as central and peripheral neurological symptoms. We report a case of a 23-year-old male presenting with severe pancytopenia, who was treated initially with red blood cell transfusions, intramuscular cyanocobalamin, and oral folate. The patient’s laboratory parameters were normal after 50 days of therapy. During the investigation, alcohol use disorder (AUD) was identified and considered to be the cause of the vitamin deficiencies once other etiologies were excluded. While these vitamin deficiencies are more common among the elderly or malnourished populations, the high prevalence of AUD in young adults, mainly in men, remains an important cause and highlights the urgent need to be addressed both in individual cases and as part of public health measures.

## Introduction

Vitamin B12 and folate deficiency is a fairly common problem among the elderly and those with at least one risk factor, such as inflammatory bowel disease [1]. Its prevalence has been on the rise with the increasing popularity of plant-based diets [2]. The most common cause of the deficiency remains an insufficient uptake, often leading to malabsorption syndromes. Although mainly associated with strictly vegan diets, it has been shown that diets with poor animal-sourced intake, such as ovo-lacto-vegetarian diets, are also linked to vitamin B12 deficiency [3]. Malabsorption is more common in the elderly, accompanied by gastric atrophy, and in those with *Helicobacter pylor*i infection. Another cause of vitamin deficiency is alcohol abuse, and the condition has long been linked to alcoholism even without the presence of malnourishment [3,4], unrelated to the uptake of the nutrients. Although the pathology is not fully understood, it has mainly been attributed to malabsorption caused by alcohol damage to the gastric mucosa, leading to chronic gastritis and consequent reduced production of intrinsic factor [5]. We report a case of severe pancytopenia attributable to megaloblastic anemia from concurrent vitamin B12 and folate deficiency in a young adult male with previously undiagnosed alcohol use disorder (AUD).

## Case presentation

The patient was a 23-year-old male patient, without any previously diagnosed diseases, who was brought to the emergency department (ED) by a friend. The patient presented with fatigue and chest pain of insidious onset for approximately four to six weeks. He was originally from India, had been living in Portugal for eight months, and denied any recent travels, contact with animals, and consumption of unpasteurized dairy products or unfiltered water. Further questioning elicited alcohol abuse, with the patient confirming daily consumption of beer and whisky, with an average daily intake of more than 20 alcohol units. When his diet was assessed, he denied consumption of beef and pork, but reported consuming other types of meat and other animal-based products.

Upon admission, the patient's physical examination was positive for hepatomegaly with a regular border, palpable, 3 cm below the ribcage, pallor of the mucous membranes, and icteric sclerae. The neurological examination was within normal limits, with no ataxia, confusion, or sensory loss. The vital signs were stable, with a blood pressure of 115/63 mmHg and a heart rate of 110 beats/minute, consistent with mild tachycardia. The presence of excess body weight was noted.

**Figure 1:**
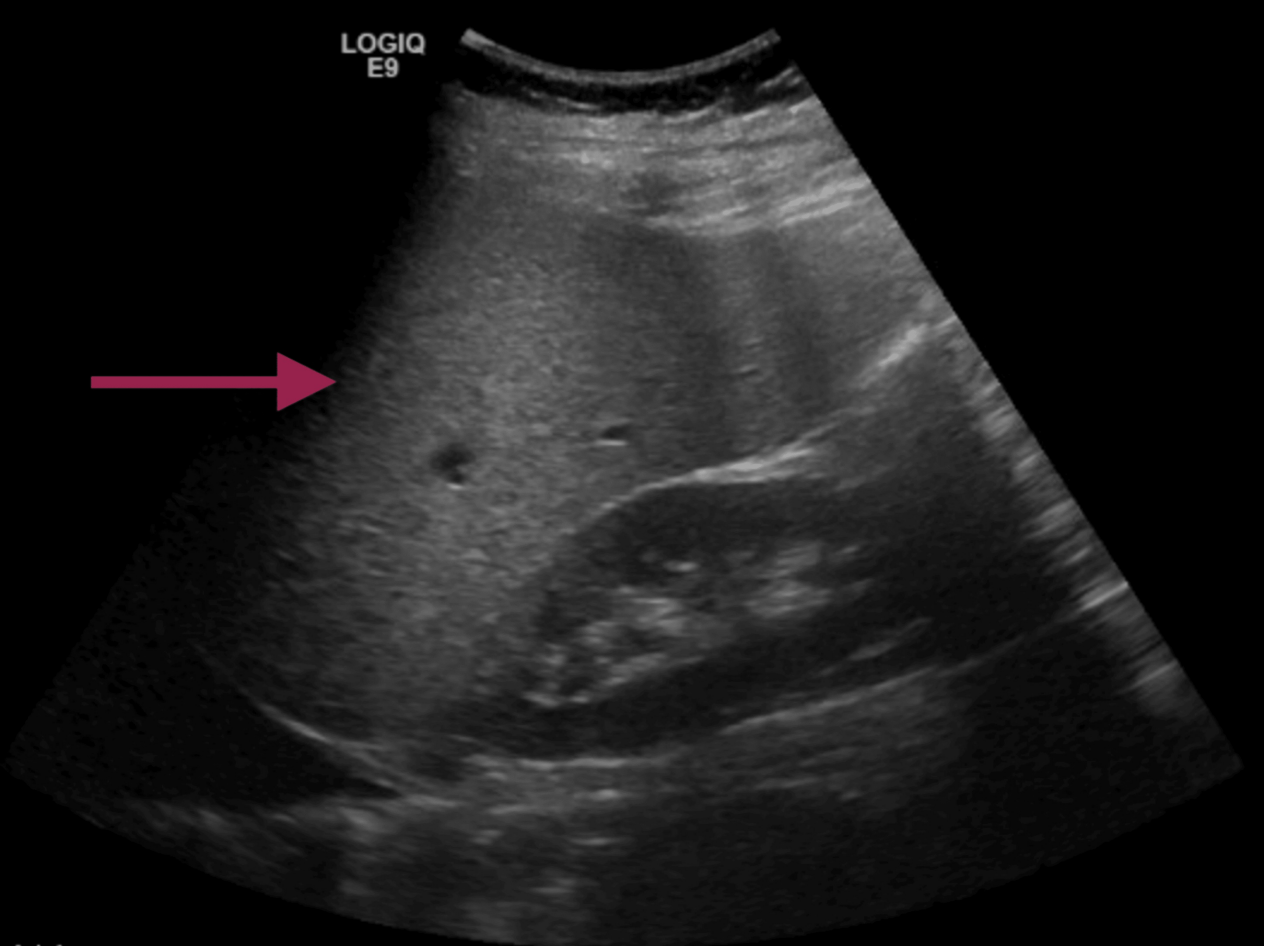
Abdominal ultrasound performed in the emergency room The image revealed homogeneous liver parenchyma with diffuse infiltrative steatosis (arrow)

Initial blood work (Table 1) was positive for severe macrocytic anemia with hemoglobin at 1.9 x 10 g/L and a mean corpuscular volume of 103.7 fL, neutropenia at 0.67 x 10^9^/L leading to leucopenia, thrombocytopenia at 11 x 10^9^/L, elevated lactate dehydrogenase at 1100 U/L, and elevated bilirubin at 10.54 mg/dL. An abdominal ultrasound was also performed, revealing homogeneous liver parenchyma with diffuse infiltrative steatosis (Figure 1). Further laboratory testing (Table 1) was performed, revealing an elevated reticulocyte index of 1.84%, an elevated sedimentation rate at 140 mm/h, and undetected levels of vitamin B12, folate, and haptoglobin. Peripheral blood smear showed hypersegmented neutrophils and confirmed red blood cell macrocytosis.

**Table 1:** Relevant altered parameters on emergency department evaluation

Laboratory parameter	Patient value	Reference range
Hemoglobin	1.9 x 10 g/L	13.0 - 17.0 x 10 g/L
Mean corpuscular volume	103.7 fL	78.0 - 96.0 fL
Neutrophils	0.67 x 10^9^/L	2.0 - 8.5 x 10^9^/L
Platelets	11 x 10^9^/L	150 - 450 x 10^9^/L
Reticulocyte index	1.84%	0.5 - 1.5
Sedimentation rate	140 mm/h	<11 mm/h
Lactate dehydrogenase	1100 U/L	135 - 225 U/L
Total bilirubin	10.54 mg/dL	<1.40 mg/dL
Direct bilirubin	4.52 mg/dL	<0.50 mg/dL
Folate	<0.6 ng/mL	3.1 - 17.5 ng/mL
Vitamin B12	<100 pg/mL	197 - 771 pg/mL
Haptoglobin	<0.05 g/L	0.30 - 2.00 g/L

The patient received immediate care with red blood cell (RBC) transfusions and a platelet transfusion; he was started on parenteral cyanocobalamin and oral folate, and was admitted to inpatient care for further investigations. He received a total of five RBC transfusions, with a post-transfusional control hemoglobin of 7.1 x 10 g/L, after which no more transfusions were required as the hemoglobin value rose slowly once cyanocobalamin and folate were administered. The neutropenia resolved after seven days of vitamin administration, with the platelet level rising progressively to a normal level after 10 days of therapy.

During the hospital stay, other causes for hemolytic anemia were excluded (Table 2), namely sickle cell anemia, thalassemias, and autoimmune hemolytic anemia. The Coombs test (Table 2) returned a weak positive result, and after consulting with Hematology, it was deemed a likely false positive because of the high prevalence of positives in hospitalized patients (7-8%) and consequent low predictive value of this test in these patients (1.4%) [6]. With the exclusion of other causes of hemolysis and its resolution after the resolution of the pancytopenia, it was deemed to be linked to immature red blood cell hemolysis secondary to the severe anemia. It was suspected that the most likely cause of all blood work alterations was the severe vitamin deficiency of both folate and vitamin B12. An endoscopy was requested to rule out atrophy of the gastric mucosa, *Helicobacter pylori* infection, or gastric cancer as possible causes of vitamin B12 deficiency, with the biopsy showing only mild gastritis. The patient also tested negative for anti-intrinsic factor antibodies and anti-parietal cell antibodies (Table 2), and hence, pernicious anemia was ruled out. 

**Table 2:** Laboratory parameters during inpatient investigation HPLC: high-performance liquid chromatography

Laboratory parameter	Result
Hemoglobin fraction quantification using HPLC	HbA: 97.2 %; HbF: 1.2 %; HbA2: not detected. Conclusion: No abnormal fractions detected
Polyspecific anti-human globulin test (Coombs test)	Weak positive
Anti-parietal cell antibodies	Negative
Anti-intrinsic factor antibodies	Negative

The patient was discharged with follow-up appointments scheduled and advised to continue treatment with cyanocobalamin. At a follow-up 50 days after diagnosis and initiation of treatment, a total resolution of all hematologic alterations was observed, with a hemoglobin of 13.8 x 10 g/L, neutrophils of 6.11 x 10^9^/L, and platelets of 339 x 10^9^/L. Changes in hematologic parameters over time are presented in Table 3.

**Table 3:** Changes in hematologic parameters over time

Parameter	Day 0	Day 1	Day 3	Day 6	Day 9	Day 50
Hemoglobin	1.9 x 10 g/L	4.2 x 10 g/L	7.1 x 10 g/L	7.7 x 10 g/L	9.0 x 10 g/L	13.8 x 10 g/L
Mean corpuscular volume	103.7 fL	88.5 fL	90.1 fL	96.7 fL	101.7 fL	87.0 fL
Neutrophils	1.88 x 10^9^/L	1.13 x 10^9^/L	0.52 x 10^9^/L	0.89 x 10^9^/L	5.25 x 10^9^/L	6.11 x 10^9^/L
Platelets	11 x 10^9^/L	9 x 10^9^/L	11 x 10^9^/L	28 x 10^9^/L	181 x 10^9^/L	339 x 10^9^/L

## Discussion

Macrocytic anemia is one among the several manifestations of severe folate and vitamin B12 deficiency, which can lead to permanent neurological damage, both central, causing dementia and behavioral changes, and peripheral, causing sensory and eventually motor neuropathy. Given its significance for both neurologic and hematologic systems, it is imperative to find the etiology of vitamin B12 deficiency to maintain its levels after diagnosis, as some causes may require periodic parenteral administrations or lifelong high-dose oral medication [7,8]. Folic acid, the synthetic form of folate, is more bioavailable [9], with oral medication being effective. This nutrient deficiency is most common in the elderly and in patients with an underlying cause of malabsorption; however, when such factors are absent, it is important to look for a different cause. In younger patients such as ours, with a lower probability of gastric atrophy, and when autoimmune causes and restrictive dietary regimens have been ruled out, it is important to address the possibility of alcohol abuse as a cause.

According to the 2024 World Health Organization report [10], the global prevalence of alcohol abuse was 7% in the population aged 15 years and older. It notes that males have a higher prevalence of heavy continuous drinking, standing at 6.7% when compared to women at 0.6%. It is also important to note that the same report states that the highest proportion of alcohol-attributed deaths was among people aged 20-39 years [10]. Given the high prevalence of alcohol use disorders among young adults, it is clinically essential to consider this as a cause for folate and vitamin B12 deficiency [3], to address both problems and prevent poor patient outcomes.

## Conclusions

We discussed a case of a young and overweight patient with severe macrocytic anemia, who presented with hemolysis accompanied by neutropenia and thrombocytopenia. It was attributed to the deficiency of folate and vitamin B12 after other causes of hemolysis were excluded, and the patient was treated successfully with parenteral cyanocobalamin and oral folate administration. Obtaining a complete clinical history enabled the identification of chronic alcohol consumption, which has long been linked to the deficiency of these two nutrients and consequent megaloblastic anemia. Our findings highlight that while excessive and chronic alcohol consumption is rarely associated with younger adults, AUD seems to have more severe consequences in this age group, and identifying both the AUD and the specific cause of vitamin deficiency is crucial to ensure favorable patient outcomes.
